# Isolation, Physicochemical Characterization, and Biological Properties of Inotodiol, the Potent Pharmaceutical Oxysterol from Chaga Mushroom

**DOI:** 10.3390/antiox12020447

**Published:** 2023-02-10

**Authors:** Phu Cuong Nguyen, My Tuyen Thi Nguyen, Ba Tai Truong, Dae-Ryeol Kim, Sujin Shin, Ju-Eun Kim, Kyu-Been Park, Ji-Hyun Park, Phuong Lan Tran, So-Young Ban, Jaehan Kim, Jong-Tae Park

**Affiliations:** 1 Department of Food Science and Technology, Chungnam National University, Daejeon 34134, Republic of Korea; 2 Department of Food and Nutrition, Chungnam National University, Daejeon 34134, Republic of Korea; 3 Department of Food Technology, Can Tho University, Can Tho 94000, Vietnam; 4 Graduated School of Energy Science and Technology, Chungnam National University, Daejeon 34134, Republic of Korea; 5 CARBOEXPERT Inc., Daejeon 34134, Republic of Korea; 6 Department of Food Technology, An Giang University, Long Xuyen 880000, Vietnam; 7 Vietnam National University of Ho Chi Minh City, Ho Chi Minh 700000, Vietnam

**Keywords:** inotodiol, solubility in organic solvents, melting temperature, microemulsion, pharmacokinetics, acute sepsis

## Abstract

Inotodiol, an oxysterol found only in Chaga mushroom, has received attention from the pharmaceutical industry due to its strong antioxidant and anti-allergic activities. However, the production of inotodiol is still challenging, and its fundamental properties have yet to be investigated. This study aims to develop an efficient method to produce high-purity inotodiol from Chaga mushroom. Then, pure inotodiol was used to assess its physicochemical properties and biological activities. By optimizing the solvent used for extraction and purification, a new method to produce inotodiol was developed with high purity (>97%) and purification yield (33.6%). Inotodiol exhibited a melting point (192.06 °C) much higher than lanosterol and cholesterol. However, the solubility of inotodiol in organic solvents was notably lower than those of the other two sterols. The difference in the hydroxyl group at C-22 of inotodiol has shown the distinctive physicochemical properties of inotodiol compared with cholesterol and lanosterol. Based on those findings, a nonionic surfactant-based delivery system for inotodiol was developed to improve its bioavailability. The inotodiol microemulsion prepared with 1–2% Tween-80 exhibited homogenous droplets with an acceptable diameter (354 to 217 nm) and encapsulation efficiency (85.6–86.9%). The pharmacokinetic analysis of inotodiol microemulsion in oral administration of 4.5 mg/kg exhibited AUC_0–24h_ = 341.81 (ng·h/mL), and C_max_ = 88.05 (ng/mL). Notably, when the dose increased from 4.5 to 8.0 mg/kg, the bioavailability of inotodiol decreased from 41.32% to 33.28%. In a mouse model of sepsis, the serum level of interleukin-6 significantly decreased, and the rectal temperature of mice was recovered in the inotodiol emulsion group, indicating that inotodiol microemulsion is an effective oral delivery method. These results could provide valuable information for applying inotodiol in functional food, cosmetic, and pharmaceutical industries.

## 1. Introduction

Inotodiol, an oxysterol found only in Chaga mushroom (*Inonotus obliquus*), attracts great interest from the food and pharmaceutical industries due to its potent biological activities. It has been reported to have remarkable biological properties in terms of anticancer [1,2,3], antioxidant [4], anti-aging [5], anti-inflammatory [6], and anti-allergic properties [7,8]. In particular, in a mouse model of food allergy, oral administration of inotodiol prevents anaphylaxis and the recurrence of food allergy symptoms [8]. Inotodiol selectively stabilizes and inhibits the activity of mast cells which modulate immune responses in various circumstances among different immune cells. When the mast cells are suppressed by inotodiol, the release of mast cell mediators such as histamine, tryptases, and various cytokines is prevented. Unlike corticosteroids, inotodiol might not increase the susceptibility to infectious disease [9]. Therefore, developing functional food and drugs using inotodiol could provide great benefits to human health in preventing and treating allergic diseases. 

**Figure 1:**
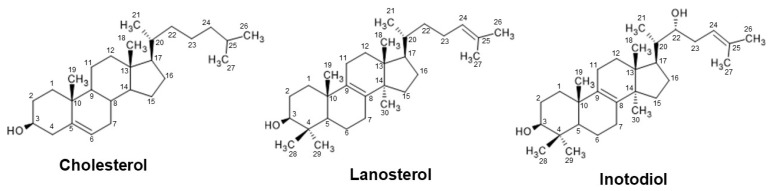
The structures of cholesterol, lanosterol, and inotodiol.

Inotodiol has a structure similar to that of lanosterol (Figure 1), and it is believed that inotodiol is derived from lanosterol through sterol biosynthesis in the Chaga mushroom. Both sterols contain the same core skeleton of cholesterol and unsaturated side chain but possess a different hydroxyl group. Like cholesterol, lanosterol has the only hydroxyl group at C-3, whereas the hydroxyl groups of inotodiol are located at C-22 and C-3. Although the difference in the structure of sterols is very little, each molecule has specific properties that distinguish them from each other. In previous studies, triterpenes and steroids in Chaga mushrooms that have structures with a hydroxyl group at C-22 and an unsaturated bond in the side chain showed stronger biological activities than other sterols. Among them, inotodiol exhibited the highest biological activity [10].

Although the biological activities of inotodiol have been widely studied, its production and purification strategies are still challenging. Inotodiol is found only in Chaga mushrooms at a low concentration (>0.2%) and has low solubility in water [7]. Inotodiol has been extracted from Chaga mushrooms using organic solvents such as ethanol, dichloromethane, and petroleum ether [4,7,8]. To obtain a high yield and separation efficiency by selecting the optimal solvent, knowledge of the solubility of inotodiol in various solvents is a prerequisite. Furthermore, to the best of our knowledge, the physicochemical properties and bioavailability of inotodiol, such as solubility and thermal behavior, have not been fully understood. These are essential properties that must be deeply understood when one wants to use inotodiol for drug, functional foods, and cosmetics development.

The objectives of this study were: (i) To establish an efficient purification process of inotodiol from Chaga mushrooms. This method could produce large amounts of pure inotodiol (>99%), providing enough inotodiol for industrial applications on larger scales. (ii) To evaluate the solubility and thermal behavior of inotodiol by comparing with two other common sterols (cholesterol and lanosterol) in combined studies in vitro and in silico. (iii) To investigate the pharmacokinetics and anti-sepsis properties of inotodiol in vivo. These results could provide valuable information for its application in the food, cosmetic, and pharmaceutical fields.

## 2. Materials and Methods

### 2.1. Materials and Reagents

The Chaga mushroom powder was purchased from Jungwoodang Co., Ltd. (Seoul, Republic of Korea). The organic solvents, including acetone, acetonitrile, dichloromethane, ethanol (food grade), ethyl acetate, methanol, and *n*-hexane, were purchased from Samchun Pure Chemical Co., Ltd. (Gyeonggi-do, Republic of Korea). Inotodiol (>98%) was purchased from Wuhan ChemFaces Biochemical Co., Ltd. (Wuhan, China). Lanosterol (>95%) and cholesterol (>95%) were purchased from Merck (Darmstadt, Germany). CDCl_3_ (99.9%), used for NMR analyses, was obtained from Eurisotop (Saint Aubin, France). All the other chemicals used were of analytical grade.

### 2.2. Inotodiol Extraction and Purification

First, 500 g of Chaga mushroom powder was mixed with 6 L of food-grade ethanol in a 10 L jar reactor (LiFlus GX, Biotron, Bucheon, Republic of Korea). The mixture was then incubated at 50 °C overnight with agitation (200 rpm). Next, the extracted solution was separated from the insoluble fraction by centrifugation (12,400× *g*, 20 °C, 20 min). Then, the insoluble residue was treated twice again with the same method to increase the yield of inotodiol. Finally, all the supernatants were collected and concentrated to a small volume (50–70 mL) with a vacuum rotary evaporator (Eyela, Tokyo, Japan).

For inotodiol purification, the concentrated extract was incubated at 60 °C for 10 min in an ultrasonic water bath (Power sonic 410, Hwashin, Seoul, Republic of Korea) to maximize the compounds’ solubility. Subsequently, 40 mL of the supernatant obtained after centrifugation (20,000× *g*, 20 °C, for 20 min) was subjected to a preparative HPLC system (LC-Forte/R, YMCKOREA) equipped with a glass column (50 × 500 cm) packed with ODS-AQ C18 (YMCKOREA, Seongnam, Republic of Korea) at a flow rate of 30 mL/min. The mobile phase was distilled water (solvent A) and methanol (solvent B), with the following gradient elution program: (0–30 min; 90–92% B), (30–45 min; 92–100% B), (45–80 min; 100% B). The effluent was continuously monitored using UV absorbance at 210 nm. The elution was fractionated according to the chromatogram that appeared. All fractions containing inotodiol were identified according to the description provided in Section 2.3.1, and the purity of inotodiol was calculated based on the area normalization method. Finally, the high-purity inotodiol (>90%) was collected and dried to a powder using a freeze dryer (MCFD, Ilshin Biobase, Dongducheon, Republic of Korea).

### 2.3. Analytic Methodology

#### 2.3.1. High-Performance Liquid Chromatography Evaporative Light-Scattering Detector (HPLC-ELSD)

In this study, the identification and quantitative determination of inotodiol, cholesterol, and lanosterol were performed using a Waters 1525 Binary HPLC system (Waters, Milford, MA, USA) equipped with an evaporative light-scattering detector (Alltech 2000, Deerfield, IL, USA) and a YMC-triart C18 column (4.6 mm × 250 mm, YMCKOREA, Seongnam, Republic of Korea). The mobile phase gradients and instrument conditions were presented in Table S1 of Supplementary Data S1. All samples were run in replicates, with each being repeated at least three times.

#### 2.3.2. High-Performance Liquid Chromatography-Tandem Mass Spectrometry (HPLC-MS/MS)

The concentration and purity of inotodiol were identified using an Agilent-1290 UPLC/6470A, and an Agilent-1290 UPLC/6430A QQQ system (Agilent Technologies Korea, Seoul, Republic of Korea), equipped with an electrospray ionization (ESI) source and the Zorbax Eclipse Plus C18 column (2.1 × 50 mm, 3.5 µm) (Agilent Technologies Republic of Korea). The mobile phase consisted of solution A (distilled water) and solution B (methanol), containing 5 mM of ammonium acetate and 0.1% formic acid in each solvent. The mass spectrometer was run in positive mode with the mobile phase gradients, and instrument conditions were presented in Tables S2 and S3 of Supplementary Data S2. The purity of inotodiol was identified using the scan mode with a mass range from 300 to 1000 *m*/*z*. The concentration of inotodiol in mouse plasma was analyzed using the MRM mode. The standard curve was prepared in the range of 1–100 ppb. The limits of detection (LOD) and quantitation (LOQ) were 1 and 3 ppb, respectively. All samples were run in replicates, with each being repeated at least three times. 

#### 2.3.3. Nuclear Magnetic Resonance (NMR)

For structural analysis of inotodiol, ^1^H-NMR and ^13^C-NMR were conducted. First, the purified inotodiol (5 mg) was dissolved in 0.7 mL of CDCl_3_. Then, ^1^H-NMR and ^13^C-NMR spectra of inotodiol were recorded at 25 °C on a Bruker Avance-III-600 (Bruker Korea, Seongnam, Republic of Korea) at the frequencies of 600.23 MHz and 150.93 MHz, respectively. Data were processed using the academic version of the TopSpin™ 4.0 software (Bruker).

### 2.4. Physicochemical Properties

#### 2.4.1. Thermal Analysis

The thermal properties of inotodiol, cholesterol, and lanosterol were analyzed using a differential scanning calorimeter (DSC-1, Mettler Toledo, Greifensee, Switzerland). Two milligrams of each dried sample were placed in a sealed aluminum pan for measurement. The heating rate was 10 °C/min in a temperature range of 25 to 200 °C.

#### 2.4.2. Solubility Analyses in Organic Solvents 

The solubility of inotodiol in various organic solvents was determined as in the description of Nguyen et al. with slight modifications [11]. First, an excess amount of inotodiol powder (50 mg, purity > 90%) was added into 1 mL of different solvents (acetone, acetonitrile, dichloromethane, ethanol, ethyl acetate, methanol, and *n*-hexane). Subsequently, the mixtures were treated with an ultrasonic water bath (Power sonic 410) for 30 min. Then, the mixtures were incubated at 30 °C for 3 h. The supernatant obtained after centrifugation (20,000× *g*, for 10 min) was filtered through a 0.2 μm membrane filter (DISMIC 13HP020AN, ADVANTEC, Tokyo, Japan) to remove any undissolved inotodiol crystals. Inotodiol determination was conducted with a HPLC-ELSD system (Alltech 2000) according to the description in Section 2.3.1. Finally, the inotodiol concentration was calculated using an established standard curve (0–1 mg/mL, R^2^ = 0.9995). The solubilities of cholesterol and lanosterol were determined with the same above method.

To understand the effect of temperature on the solubility of inotodiol, inotodiol was dissolved at different temperatures, ranging from 10 °C to 50 °C in ethanol, methanol, acetonitrile, acetone, or *n*-hexane, using the same above method. All experiments were performed in duplicate. 

#### 2.4.3. Density Functional Theory (DFT) Calculations 

All DFT calculations were performed using the Gaussian 16 [12] package. The isolated structure of inotodiol, cholesterol, and lanosterol was optimized at the wB97XD/6–311G (d,p) level of theory by using gas phase solvation SMD models [13]. Several different solvents, including acetone, acetonitrile, dichloromethane, ethanol, methanol, and *n*-hexane, were used.

### 2.5. Inotodiol Emulsion Study

#### 2.5.1. Inotodiol Emulsion Preparation

The formulation of oil in water (O/W) emulsions of inotodiol was prepared according to the previous studies’ procedure with some modifications [14,15]. Briefly, the oil phase and aqueous phase were separately treated. First, in the oil phase, the calculated amount of inotodiol (>97%) was mixed in the olive oil (10% *v*/*v*, in the final emulsion). The aqueous phase consisted of Tween-80 (0.1, 0.5, 1, and 2% *w*/*v*, in the final emulsion) and distilled water. Then, these mixtures were treated with an ultrasonic water bath (Power sonic 410) for 30 min. Subsequently, the aqueous phase was gradually added to the oil phase. Then, the components were further vortexed for 5 min and then subjected to ultrasonication (Sonicator ultrasonic processor, Qsonica; Newtown, CT, USA) at an amplitude of 70, with a pulse of 10 s (on) and 5 s (off) for 20 min to obtain a microemulsion.

#### 2.5.2. Encapsulation Efficiency (EE)

The encapsulation efficiency of the Tween-80-encapsulated inotodiol microparticles was calculated using the following equation (Equation (1)):

(1) $$\mathrm{EE}\;\left(\%\right)=\frac{C_{0}}{{\;C}_{1}{}_{\;}}\times 100$$

In the above equation, $C_{0}$ and $C_{1}$ represent the inotodiol content in the microcapsules and the total initial inotodiol added to the microcapsules, respectively.

#### 2.5.3. Microstructure, Mean Size, Polydispersity Index (PDI), and Zeta Potential Analysis

The microstructure of inotodiol emulsions was analyzed using an optical microscope (Olympus BX45, Olympus Optical Co., Tokyo, Japan) according to a previous report [15]. Briefly, 10 μL of the emulsion was placed on a microscope glass slide and covered by a coverslip. Then, the microdroplets of inotodiol emulsions were observed in an optical microscope with 100X oil immersion objective lens. 

The mean size, polydispersity index (PDI), and zeta potential of inotodiol emulsions were measured (three times in parallel) using the Zetasizer Nano ZS (Malvern Instruments Ltd., Worcestershire, UK).

### 2.6. Pharmacokinetic Study and Analysis

#### 2.6.1. Animals

All mice (male ICR mice, eight-week-old) have maintained individually ventilated cages located in a specific pathogen-free room at a constant temperature of 22 ± 1 °C, a humidity of 55 ± 10%, and a 12 h light/dark cycle. These mice were divided into three groups, and each group consisted of eight mice: groups I, II, and III. Briefly, groups I and II were oral administrations (p.o.) with different doses of 4.5 mg/kg and 8.0 mg/kg of inotodiol, respectively. Group III was intraperitoneal administration (i.p.) treated with 4.5 mg/kg of inotodiol. The microemulsions at the dosages of 4.5 and 8.0 mg/kg of inotodiol were prepared according to the method in Section 2.5.1.

Blood samples (50 μL) were collected at 0.25, 0.5, 1, 2, 4, 8, and 24 h after p.o. and i.p. administrations. The samples were immediately centrifuged at 4000 rpm, 25 °C, for 15 min to obtain plasma. Then, the plasma samples were stored in the freezer at −80 °C until further use. 

#### 2.6.2. Determination of Inotodiol Concentration in Plasma

Here, 20 µL of serum was transferred to a 1.5 mL Eppendorf tube. Then, the sample was extracted twice with ethanol. In each extraction step, 200 µL of ethanol was mixed with serum, and the mixture was sonicated for 10 min before centrifugation (14,000× *g*, for 15 min). The supernatant of the two extraction steps was mixed and ultimately evaporated. The residue was reconstituted with 0.5 mL of 60% methanol for solid-phase extraction using Sep-Pak Vac 3 cc (200 mg) C18 cartridges (Waters Corp. Milford, MA, USA). The sample was loaded into the cartridge, which was activated using methanol (6 mL) and conditioned by 60% of methanol (6 mL). The cartridge was washed with 6 mL of methanol 60%. Then, inotodiol was eluted with 100% of methanol (3 mL) in the glass tube. The sample was then evaporated. The residue was resolved in methanol before HPLC-MS/MS analysis.

#### 2.6.3. Data Analysis

The pharmacokinetic parameters of inotodiol, including elimination half-time (t_1/2_), time to reach the maximum plasma concentration (T_max_), the maximum plasma concentration (C_max_), and the area under the plasma concentration–time curve (AUC), were determined by the Microsoft add-in tool and PKSolver [16], using the first-order compartmental analysis. Oral bioavailability (F, %) was calculated from the following formula [17]:

(2) $$F=\frac{{\left[{\mathrm{AUC}}_{0-24}\right]}_{\;p.o.}/{\mathrm{Dose}}_{p.o.}}{{\left[{\mathrm{AUC}}_{0-24}\right]}_{\;i.p.}/{\mathrm{Dose}}_{i.p.}}\times 100$$

where ${\left[{\mathrm{AUC}}_{0-24}\right]}_{\;p.o.}\;\mathrm{and}\;{\left[{\mathrm{AUC}}_{0-24}\right]}_{\;i.p.}$ are the area under the plasma concentration–time curve from 0 to 24 h post-dose of oral and intraperitoneal administrations, respectively.

### 2.7. Acute Sepsis Mouse Model

For the acute sepsis experiment, 6-week-old BALB/C male mice were purchased (Samtako Bio Korea, Osan, Republic of Korea) and housed for 1 week before starting the experiment. Mice were divided into each group (4 heads for each) and allowed to eat ad libitum. Groups were designed as naïve, sham, emulsion, mixture, and dexamethasone. Emulsion or mixture states of inotodiol (4.5 mg/kg) and dexamethasone were orally administered to each group once daily for 4 days. Then, lipopolysaccharide (LPS) (5 mg/kg) was i.p. injected into mice after 2 h of the last oral administration, except for the naïve group. Blood samples were collected for measuring proinflammatory cytokines, including tumor necrosis factor α (TNF-α), interleukin-1β (IL-1β), and IL-6. In addition, the vital signs of mice were monitored, and the rectal temperature was measured before sacrifice. To measure proinflammatory cytokine concentrations of TNF-α, IL-1β, and IL-6 in serum, they were determined using an enzyme-linked immunoassay kit (ELISA kit, Biolegend, San Diego, CA, USA), following the manufacturer’s guidance.

## 3. Results and Discussion

### 3.1. Selection of the Extraction Solvent

Solvent selection is one of the crucial parameters affecting the extraction process’ efficiency. As the first step, various organic solvents with different polarities and hydrophobicities, including acetone, acetonitrile, dichloromethane, ethanol, ethyl acetate, methanol, and *n*-hexane, were evaluated in terms of solubility and safety to select the suitable solvent for inotodiol extraction. As shown in Table 1, at a fixed temperature (30 °C), inotodiol exhibited the highest solubility in ethanol and dichloromethane (24–25 mg/mL). Ethyl acetate, methanol, and acetone were able to solubilize a moderate yield of inotodiol (6–8 mg/mL). However, the solubilization yield of inotodiol in acetonitrile and *n*-hexane was poor (0.2–1 mg/mL). In addition, it was observed that the solubility of inotodiol in ethanol, methanol, and acetone significantly increased with the increasing temperature, from 10 °C to 50 °C (Figure S1 of Supplementary Data S3). In contrast, acetonitrile and *n*-hexane provided low solubilization yields of inotodiol in this temperature range. 

Overall, ethanol and dichloromethane showed the ability to solubilize a high yield of inotodiol. This finding was closely related to a previous report that the crude ethanol extract and dichloromethane fractions obtained from Chaga mushroom had intense anti-allergic activity [7]. On the other hand, regarding solubility and toxicity, ethanol was selected as the best solvent for leaching inotodiol from Chaga mushroom because it could dissolve a similar concentration of inotodiol and was less toxic than dichloromethane. In fact, dichloromethane is also used to extract some natural products from plants, but it is not recommended for pharmaceutical products due to its high toxicity [18]. Meanwhile, ethanol is considered a safe solvent, widely used in industry [19,20]. Furthermore, previous research on triterpenoids’ extraction from *Sanghuangporus sanghuang mycelium* using organic solvents proved that ethanol also provided a high solubilization yield of triterpenoids with high antioxidant activities [21]. Therefore, we chose ethanol as the ideal solvent to be used in large-scale inotodiol extraction.

**Table 1:** Solubility of inotodiol in various solvents at 30 °C.

Solvent	Relative Polarity ^(i)^	Log P ^(ii)^	Toxicity ^(iii)^	Solubility of Inotodiol (mg/mL)
Acetone	0.355	−0.23	class 3	5.70 ± 0.39
Acetonitrile	0.460	−0.33	class 2	1.11 ± 0.19
Dichloromethane	0.309	0.60	class 2	24.00 ± 0.95
Ethanol	0.654	−0.24	class 3	24.84 ± 0.43
Ethyl acetate	0.228	0.68	class 3	8.18 ± 0.71
Methanol	0.762	−0.76	class 2	8.13 ± 0.24
*n*-Hexane	0.009	3.50	class 2	0.15 ± 0.07

^(i)^ The values for relative polarity were taken from Christian Reichardt [22], considering water as a reference (polarity = 1). ^(ii)^ The log P values that are the partition coefficient’s logarithms express the hydrophobicity of solvents and are taken from previous studies [23,24]. ^(iii)^ According to the U.S Food and Drug Administration [18].

### 3.2. Large-Scale Extraction and Purification of Inotodiol

**Figure 2:**
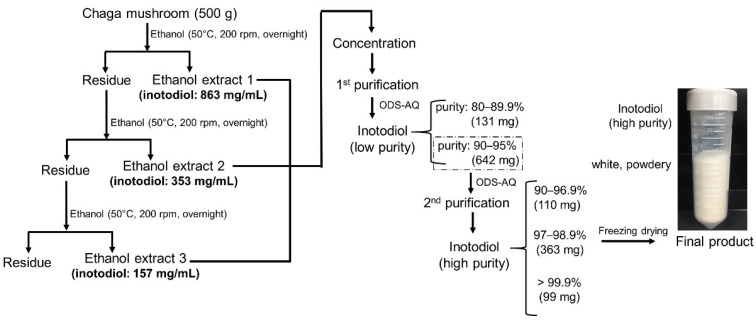
Schematic diagram of inotodiol purification. Using a simple and efficient process, high-purity inotodiol (>97%) was obtained with a 33.6% purification yield from the crude extract.

The extraction and purification procedures of inotodiol from Chaga mushroom are shown in Figure 2. The extraction amounts of inotodiol in the second and third ethanol extract stage decreased to 2.4- and 5.5-fold, respectively, compared with the preceding stage. As a result, the total amount of inotodiol in the ethanol extractions was approximately 1.4 g, and the yield accounted for 0.28% (*w*/*w*). This process achieved a higher extraction yield than a previous method, which obtained only 0.1% of inotodiol yield from 3 extractions with 70% ethanol in water [7]. The low water solubility of inotodiol could result in the solubilization yield of inotodiol in 70% ethanol being lower than in ethanol. Moreover, in another study, the supercritical fluid extraction and Folch methods were also applied for inotodiol extraction, but the extraction yield obtained only 0.1% and 0.14%, respectively [25].

In order to obtain the high purity of inotodiol from the Chaga extract, in this work, we used reverse-phase chromatography, which is a highly effective separation system for pure substances [26]. First, we screened a suitable mobile phase for separating inotodiol using the reverse-phase column. Commonly, acetonitrile and methanol with water are recommended as a mobile phase for reverse-phase chromatography [27]. Therefore, using this mobile phase, we have briefly tested the separation of inotodiol (0.5 mg/mL) on the commercial analysis C18 column. As shown in Figure S2 (Supplementary Data S4), acetonitrile and methanol could elute inotodiol with sharp peaks during the same gradient condition. However, the peak area of inotodiol eluted by methanol was 3-fold greater than inotodiol eluted by acetonitrile. Additionally, the inotodiol peak was shifted to an earlier retention time when methanol was used for the elution, indicating that methanol’s ability to separate inotodiol was better than acetonitrile. These findings are consistent with the results of inotodiol solubility in methanol being higher than in acetonitrile (Table 1).

Methanol was chosen as a mobile phase for the large-scale inotodiol purification from the results. To obtain pure inotodiol, we performed two chromatographic steps in this work. First, the crude inotodiol was fractionated using a preparative HPLC system with the reverse-phase chromatography column. After identifying the inotodiol fractions by analytical HPLC, the obtained inotodiol was divided into two main groups, which contained medium purity (80–89.9%) and high purity (>90%). It has been observed that the purification yield of inotodiol (purity > 90%) derived from this method (46.8%, *w*/*w*) was much higher than that of inotodiol produced from the previous method (3.5%, *w*/*w*), which used *n*-hexane and ethyl acetate (20:1) as a co-solvent for the elution of inotodiol in the reverse-phase chromatography [7]. The reason is probably due to the lower solubility of inotodiol in *n*-hexane, resulting in a poor yield. Overall, the extraction and purification yields of inotodiol in this work were much improved compared to the previous studies due to the selection of suitable solvents.

In general, the high purity of inotodiol (90–95%) obtained in the first step could meet the requirements for some specific applications. For pharmaceutical studies, however, it has been further purified using the preparative HPLC system under the same condition to obtain the highly pure inotodiol. It was observed that the purity of inotodiol in the second purification was greatly improved. The highest purity even reached a greater 99%, suitable for use in many laboratory and analytical applications. The fractions containing inotodiol were collected and divided into three main groups, having different purities of 90–96.9%, 97–98.9%, and 99–99.9%, with the purification yields of 17.1%, 56.5%, and 15.4%, respectively.

In summary, utilizing suitable solvents for extraction and purification produced inotodiol with a significant yield. The two-step purification method produced high-purity inotodiol (>97%) with a 33.6% purification yield from the crude extract. Moreover, this method produced inotodiol with much higher purity and yield (purity > 97%, 924 mg inotodiol from 1 kg Chaga mushroom powder) than the method in the previous study (purity > 95%, 330 mg from 1 kg Chaga mushroom powder) [7]. Therefore, this procedure effectively extracts and purifies inotodiol from Chaga mushrooms with good prospects. Taken together, it was demonstrated that our purification strategy was adequate for the isolation of inotodiol.

### 3.3. Structure Characterization

**Figure 3:**
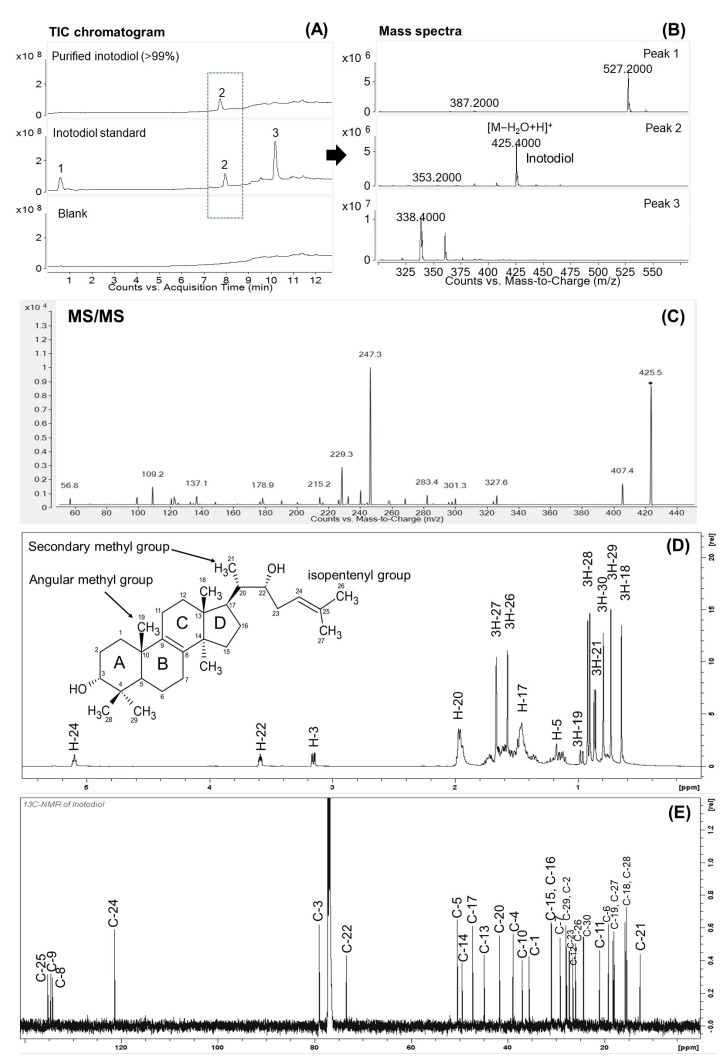
(**A**) Total ion chromatogram (TIC). (**B**) Mass spectra of inotodiol. (**C**) The MS/MS spectra of inotodiol. (**D**) ^1^H and (**E**) ^13^C NMR spectra of inotodiol.

After the purification, the pure inotodiol (>99%) was subjected to HPLC-MS/MS analysis to confirm its purity and structure. Additionally, the purified inotodiol was further validated by comparing its retention time and mass spectra with the inotodiol standard. As shown in the total ion chromatogram (TIC) (Figure 3A), the purified inotodiol showed only one peak at the retention time of 8.2 min, while three major peaks were observed in the TIC of the commercial standard. The mass spectra of the peak at 8.2 min of the retention time (peak 2) presented the [M−H_2_O+H]^+^ ion at m/z 425 (Figure 3B), which corresponded to the expected molecular mass for inotodiol, 442.7 g/mol. The MS/MS spectra of peak 2 (Figure 3C) showed the major fragment ions at m/z 109, 229, 247, 259, and 407, and the proposed fragmentation pathways were shown in Figure S3. Tentatively, the ion at m/z 407 was produced by eliminating the water molecule from the ion m/z 425. Besides, the formation of the fragment ions at m/z 247 and 229 were probably produced by the dissociation within the ring C with the characteristic retro-Diels–Alder (RDA) cleavage. As reported, the RDA dissociation of ring C of stanols and lanosterol produced fragment ions at the highest intensity among all fragments [28]. As seen in the MS/MS spectra, the most abundant fragment ion was m/z 247, indicating that the RDA dissociation of ring C of inotodiol occurred. This observation agrees with the findings of Géry [29], who reported that the MS/MS of inotodiol produced the fragment ion at m/z 247 predominantly when the collision energy and fragment voltage were set at 12 V and 100 V, respectively. Based on the MS/MS spectra and the proposed fragmentation pathways, peak 2 was identified as inotodiol. Additionally, the mass spectra of peaks 1 and 3 of the inotodiol standard showed the molecular ions at m/z 527.2 and 338.4, respectively. These impurities could be inotodiol derivatives. The results indicated that the purified inotodiol reached high purity, even better than the commercial standard. Therefore, the obtained inotodiol in this study could be used for subsequent experiments. Furthermore, its quality could meet strict requirement standards in some industries.

The chemical structure and purity of inotodiol were also analyzed by ^1^H and ^13^C NMR. The assigned data were provided in the Supplementary Data S6. As shown in the ^1^H and ^13^C NMR spectra of inotodiol (Figure 3D,E), the signals related to five angular methyl groups (δ_H_ 0.64 (s, H3-18), 0.73 (s, H3-29), 0.79 (s, H3-30), 0.91 (d, H3-28), 0.97 (dd, H3-19)), one secondary methyl group (δ_H_ 0.86 (d, 3H-21)), an olefinic group between C-8 (δ_C_ 134.58) and C-9 (δ_C_ 134.20), and an isopentenyl group in the side chain (δ_H_ 1.57 (s, H3-26), 1.66 (s, H3-27)), (δ_H_ 5.10 (t, H-24), δ_C_ 121.25 (C-24)), indicated the presence of a lanostane-type triterpenoid structure. An oxygenated methine group at C-3 (δ_H_ 3.15 (dd, 3-H), δ_C_ 78.98) was also observed. Hence, its structure was similar to lanosterol [30], except for the presence of an additional oxygenated methine group at C-22 (δ_H_ 3.59 (m, 22-H), δ_C_ 73.38). These results indicated that the presence of two hydroxyl groups at C-3 and C-22 in the target molecule was assigned as inotodiol. This observation is consistent with the findings in previous studies [3,31]. Additionally, no impurity peak was observed on the ^1^H and ^13^C NMR spectra of inotodiol, indicating that pure inotodiol was obtained in this study.

### 3.4. Physicochemical Properties of Inotodiol in Comparison with Cholesterol and Lanosterol

To better understand the physicochemical properties of inotodiol and how its properties differ from other analogs, this work compared the thermal behavior and solubility of inotodiol, cholesterol, and lanosterol by combining an experimental and theoretical approach.

#### 3.4.1. Thermal Property

**Figure 4:**
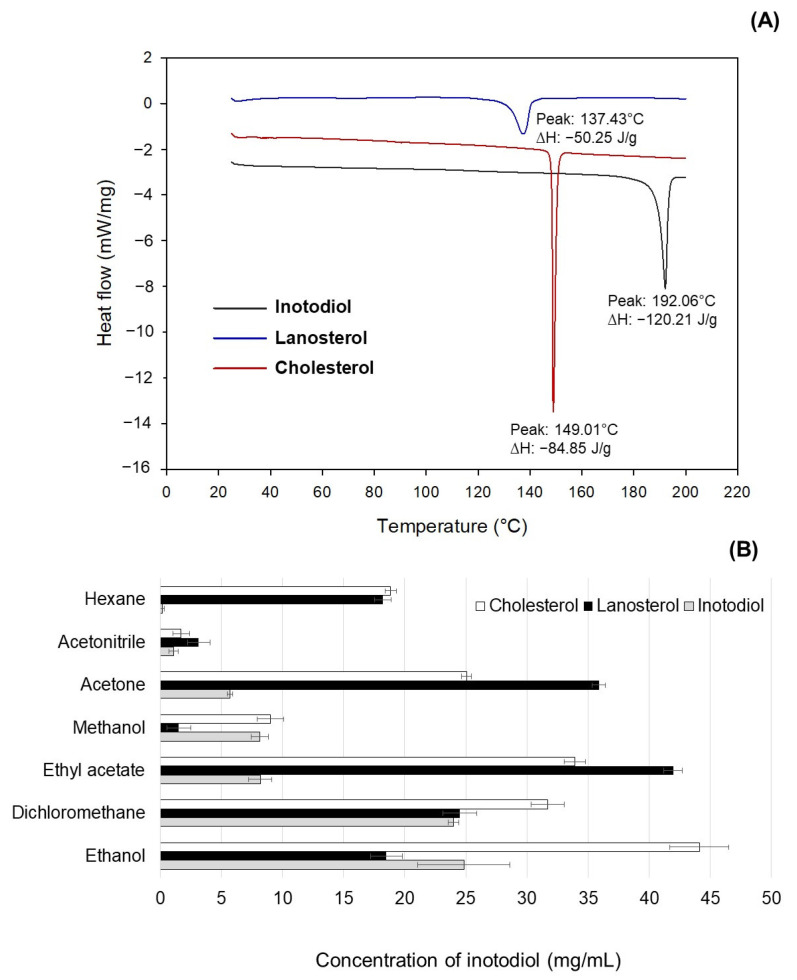
(**A**) The thermal properties of inotodiol, lanosterol, and cholesterol analyzed by differential scanning calorimetry. (**B**) The solubility of inotodiol, lanosterol, and cholesterol in various organic solvents.

The thermal behaviors of inotodiol, lanosterol, and cholesterol are presented in Figure 4A. The inotodiol showed the highest melting endotherm at 192.06 °C, while those of lanosterol and cholesterol were much lower at 137.43 °C and 149.01 °C, respectively. This finding is consistent with previous studies on lanosterol and cholesterol [32,33]. Typically, thermal decomposition requires heat absorption to break the bonds between molecules, which results in an increased melting temperature and endothermic energy (enthalpy, ΔH). Intriguingly, despite lanosterol and inotodiol sharing the backbone structure (Figure 1), the melting endotherm of inotodiol was significantly higher than that of lanosterol (Figure 4A), suggesting that inotodiol molecules were attracted to one another much more strongly than were lanosterol molecules. It is feasible that the hydroxyl group at C-22, the only structural difference of inotodiol from lanosterol, can provide stronger hydrogen bond interactions when inotodiol stacks in a crystal state. On the other hand, cholesterol and lanosterol have the same hydroxyl group at C-3 and the same backbone structure, but their thermal behaviors are not considerably different. The results suggested that inotodiol had a higher thermal stability than cholesterol and lanosterol because of the difference in the C-22 hydroxyl group.

Regarding bioavailability, compounds with lower melting temperatures are more likely to be well-absorbed than higher melting ones for any given dose [34]. Therefore, the higher thermostability of inotodiol could cause more difficulties during absorption in the human body. However, the pharmacokinetics and bioavailability of inotodiol should be determined to further confirm this hypothesis.

#### 3.4.2. Solubility in Organic Solvents

The solvents were classified into three groups according to their polarity and hydrogen-bonding capacity, including polar protic solvents (ethanol, methanol), polar aprotic solvents (acetone, acetonitrile, dichloromethane, ethyl acetate), and a non-polar solvent (*n*-hexane). The solubilities of inotodiol, lanosterol, and cholesterol in various organic solvents are presented in Figure 4B.

Overall, inotodiol showed lower solubility than lanosterol and cholesterol in all the solvents except methanol. For instance, the solubility of cholesterol in *n*-hexane, acetone, ethyl acetate, ethanol, and acetonitrile was higher than that of inotodiol, which was 129.7-, 4.4-, 4.1-, 1.8-, and 1.5-fold, respectively. Additionally, lanosterol exhibited higher solubility than inotodiol in *n*-hexane, acetone, ethyl acetate, and acetonitrile, which was 125.3-, 6.3-, 5.1-, and 2.8-fold, respectively. The results showed that the solubility trend of cholesterol and lanosterol was similar in the various solvents. The reason may be that cholesterol and lanosterol have the same functional group (C-3 hydroxyl group) with the same physical arrangement in their structure. Therefore, the polarity of the two molecules is almost similar, and they have equal abilities for specific interactions. The difference in the C-22 hydroxyl group may result in a distinct solubility behavior of inotodiol compared to cholesterol and lanosterol. To confirm this hypothesis, in addition to the experiments, we attempted to understand the fundamental interaction between inotodiol and different solvents based on the quantum chemical density functional theory (DFT) study. This is a powerful method to predict the electronic properties of molecules and materials due to accuracy and high computational efficiency. Previous reports have elucidated the fundamental interaction in the system containing solutes such as lignin and rutin with different solvents using a combined experiment with DFT and molecular dynamics [35,36].

#### 3.4.3. DFT Calculation of Molecular Interaction

The optimized geometry and selected bonding lengths of inotodiol obtained at the wB97WD/6-311G (d,p) level of theory employing gas phase and SMD solvation models with several different solvents were presented in Figure S5 and Table S4. It can be seen that the polarity of solvents significantly affects the bond lengths of inotodiol, especially for C3–O and C22–O bonds. The elongation of these bonds in high-polarity solvents such as methanol and ethanol was more elevated than in other solvents. For instance, the length of C–O bonds of inotodiol in methanol and ethanol increased 0.008–0009 Å and 0.006–0008 Å, respectively, compared to that in the gas phase. On the other hand, the change of these bonds in the aprotic solvents, such as acetonitrile, acetone, and dichloromethane, was around 0.003–0.004 Å. In nonpolar *n*-hexane, the length of C–O bonds of inotodiol deviated by only 0.001–0.002 Å. A similar trend was also observed in the experiments. The solubility of inotodiol was highest in the polar solvent ethanol and lowest in the non-polar *n*-hexane, while the aprotic solvents such as acetonitrile, acetone, and dichloromethane could solubilize a moderate yield of inotodiol. These results suggested that the hydroxyl groups located at C-3 and C-22 positions greatly influenced the inotodiol solubility behavior. In previous research, by combining the experiment with a detailed DFT study for the lignin model, Ponnuchamy et al. also demonstrated that the solvents’ polarity strongly affected the lignin’s hydroxyl group. This functional group was the key site for forming strong hydrogen bonding during the dissolution [35]. 

On the other hand, the change in the C3–O bond length of inotodiol, cholesterol, and lanosterol in all the solvents is the same pattern (Table S4), indicating that the effects of solvents on the C-3 hydroxyl group of these sterols are similar. In addition to the C3–O bond, there was no significant change in other bonds in both cholesterol and lanosterol molecules (Figures S3 and S4 of Supplementary Data S7). This observation agrees with the cholesterol and lanosterol solubility trend in experimental results. Intriguingly, the only structural difference between inotodiol and lanosterol was the hydroxyl group located at C-22, but the inotodiol solubility in *n*-hexane was much lower than in lanosterol. As mentioned above, the solvents likely strongly affected both the C-3 and C-22 hydroxyl groups. Therefore, the presence of the C-22 hydroxyl group may play a significant role in the distinct solubility behavior of inotodiol.

### 3.5. Characteristics of Inotodiol Emulsion

The poor water solubility and high melting point crystallinity of inotodiol could result in very low intestinal absorption. The previous study also reported the low oral bioavailability of inotodiol (0.45%) [37]. In this study, therefore, we developed microemulsion-based delivery systems of inotodiol to determine its pharmacokinetics and bioavailability. First, the inotodiol emulsions were prepared with different Tween-80 concentrations (0.1, 0.5, 1.0, 2.0% *w*/*v*) to select the proper surfactant amount for the inotodiol emulsion formulation. Then, the physical properties were evaluated by changing the particle size, polydispersity index, zeta potential, encapsulation efficacy, and morphology. 

**Figure 5:**
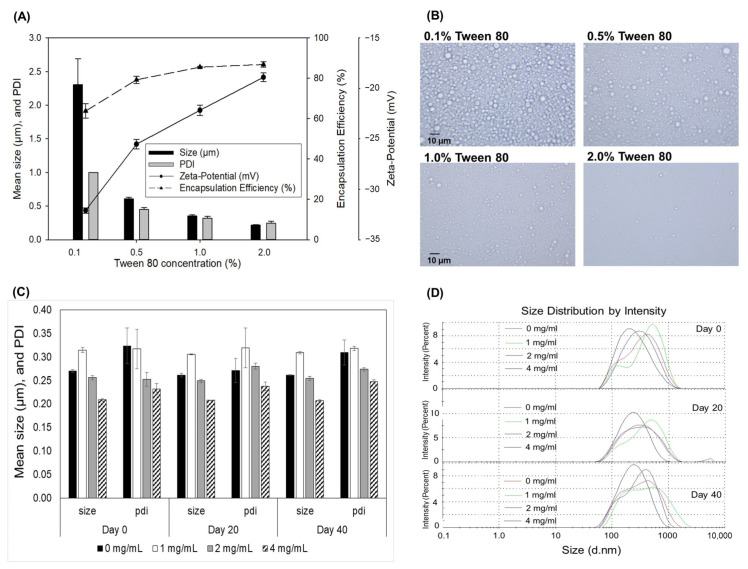
Effect of Tween-80 concentration on the size, PDI, zeta potential, and EE of inotodiol emulsion (**A**). Optical microscopy images of inotodiol emulsions stabilized with different Tween-80 concentrations (**B**). Effect of inotodiol concentration on the size, PDI (**C**), and distribution (**D**) of inotodiol emulsions during 40-day storage.

The results in Figure 5A showed that the mean droplet diameter and PDI of inotodiol emulsions gradually decreased as the Tween-80 concentration increased from 0.1% to 2% *w*/*v*. The inotodiol emulsion with 2% Tween-80 had the smallest droplet size (0.22 ± 0.44 µm) and the narrowest particle size distribution (PDI = 0.24 ± 0.03) compared with the other three concentrations. The emulsion prepared with 0.1% Tween-80 showed a broader particle size distribution (PDI = 1.0) and a bigger droplet size (2.31 ± 0.26 µm). The reason was probably due to the low Tween-80 concentration: there were insufficient Tween-80 molecules available to cover the surface of emulsion droplets, leading to the coalescence and/or flocculation of droplets, forming a bigger particle size.

On the other hand, the zeta potential of all inotodiol emulsions prepared with Tween-80 at different concentrations possessed negative values and showed a positive correlation with the Tween-80 concentration. As seen in Figure 5A, the zeta potential values increased significantly from −32.1 to −18.9 mV as the Tween-80 concentration increased from 0.1% to 2% *w*/*v*. The zeta potential of the emulsion prepared with 0.1% Tween-80 was 1.7-fold lower than that prepared with 2% Tween-80. Tween-80 is a non-ionic surfactant, and the negative charge of these droplets could arise from the presence of minority molecules adsorbed onto the interface, such as [OH]^−^ species from the aqueous phase and possibly from the inotodiol, free fatty acids, and phytosterols in the oil phase. Therefore, the higher negative charge may be due to the droplets’ surface not being wholly covered, resulting in the more attractive forces between droplets. This observation is in good agreement with the data reported in [38].

As seen in Figure 5A,B, the particle size distributions obtained using microscopy images have a similar pattern to that determined in suspension by the Zetasizer Nano ZS. At a low Tween-80 concentration, the emulsion showed the primary particles with high inhomogeneous distribution and degrees of aggregation. This could be expected because, with a further increase in the concentration of Tween-80, a higher number of Tween-80 molecules were available for covering the freshly sheared droplets during homogenization, forming smaller droplets with a more uniform distribution. Additionally, no aggregation between oil droplets was seen in the emulsion prepared with 1–2% Tween-80. Besides that, the inotodiol emulsions prepared with 1–2% Tween-80 showed a high encapsulation efficiency (85.6–86.9%), which was 1.34-fold higher than that prepared with 0.1% Tween-80 (63.8%). These results indicated that 1–2% Tween-80 is suitable for preparing the inotodiol microemulsion.

In addition, the effect of inotodiol amount on the droplet size and stability was also evaluated. As seen in Figure 5C, the mean droplet diameter and PDI of inotodiol emulsions gradually decreased as the inotodiol concentration increased from 1 to 4 mg/mL. Intriguingly, the microemulsions with an inotodiol concentration of 4 mg/mL showed droplets with more homogenous and long-term stability than other lower concentrations during 40-day storage. The results showed that a higher amount of inotodiol in the nonionic surfactant-based microemulsion system could form stable and homogenous droplets with smaller particle sizes and narrower distribution. The reason may be that inotodiol is mainly located within the hydrophobic interior of the oil droplets in the microemulsion system, and some may be located close to the oil–water interface due to the polar groups on the inotodiol. Tween-80 contains both hydrophilic and hydrophobic groups that can efficiently interact with the hydroxyl groups and the unsaturated side chain of inotodiol. They can form a potent complex through hydrogen bonds and hydrophobic interactions. A previous study observed that a direct stacking interaction occurred between cholesterol and the nonionic surfactant. The interaction strength between them was significantly affected by the number of ethylene oxide units and the carbon chain length in the surfactant molecules [39]. Therefore, encapsulating a sufficient amount of inotodiol may assist the microemulsion system to be stable against droplet growth from flocculation and coalescence.

### 3.6. Pharmacokinetics of Inotodiol

**Figure 6:**
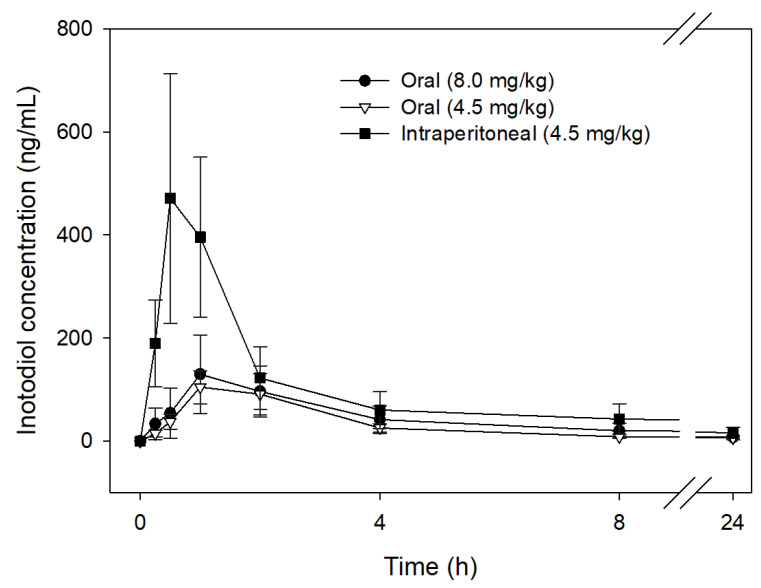
Plasma concentration profiles of inotodiol in mice after oral and intraperitoneal administration (n = 8, mean ± SD).

The low oral bioavailability of inotodiol (0.45%) and some sterols (0.04–0.5%) has been reported in previous studies [37,40]. In this work, therefore, inotodiol dissolved in olive oil was prepared as an oil in water emulsion with a 1% Tween-80 emulsifier to improve its oral bioavailability. Then, its pharmacokinetics was determined on two different routes of administration in mice (orally, intraperitoneal) at 4.5 mg/kg and 8.0 mg/kg. The concentration–time profiles of inotodiol in mouse plasma after oral and intraperitoneal administrations are shown in Figure 6, and the pharmacokinetic parameters are presented in Table 2. In the case of intraperitoneal administration (4.5 mg/kg dose), the absorption of inotodiol into plasma was speedy, and the maximum plasma concentration (C_max_) of 413 ng/mL was reached at 0.7 h. After that, the inotodiol plasma concentration abruptly decreased. It may be that inotodiol was systematically distributed and rapidly metabolized throughout the body, resulting in a short half-life, approximately 0.4 h. This finding was close to 27-hydroxycholesterol (0.75 h) after infusion in a healthy volunteer [41]. Generally, the major oxysterols in human circulation, such as 27-hydroxycholesterol and 25-hydroxycholesterol, were found to be able to pass lipophilic membranes at a high rate, 1000 times faster than cholesterol [42].

In oral administration, the plasma concentrations of inotodiol were significantly lower than those of intraperitoneal administration at all time points from 0 to 24 h (including absorption, distribution, and elimination phases) after administration. The patterns of plasma pharmacokinetic profiles of inotodiol in both groups of 8.0 mg/kg and 4.5 mg/kg were similar. After the oral administration of inotodiol at a 4.5 mg/kg dose, the maximum plasma concentration (C_max_) of 88.05 ng/mL was reached at 1.44 h, with a half-life of approximately 0.95 h. Several studies investigated the pharmacokinetic parameters of triterpene derivatives, such as betulinic acid and oleanolic acid. For instance, the C_max_ and T_max_ of betulinic acid in the plasma after the oral administration in rats at 100 mg/kg were 1.16 µg/mL and 2.36 h, respectively [43]. After the oral administration to the rat at a 25 mg/kg dose, the maximum oleanolic acid plasma concentration (C_max_) of 74 ng/mL was reached at 0.42 h [44]. The C_max_ and T_max_ values were slightly different between the studies. This may be partly due to the differences in the subject’s body weight, the dose, and the route of administration. When dose levels increased to 8.0 mg/kg, the C_max_ and T_max_ of inotodiol in the plasma were 116 ng/mL and 1.28 h, respectively, within the range of the triterpene derivatives’ values previously reported. Additionally, the AUC_0–24h_ was observed to be proportional to the dose. When 8.0 mg/kg of inotodiol was administered, the AUC_0–24h_ was 489 ng·h/mL, which was 1.42-fold higher than that of the 4.5 mg/kg dosage. It has been noticed that the variation of C_max_ and AUC_0–24h_ in the 8.0 mg/kg group was relatively high, with the relative standard deviations of 37.4% and 51.6%, respectively.

Additionally, when dose levels increased from 4.5 to 8.0 mg/kg, the bioavailability value (%F) decreased from 41.32% to 33.28% after the oral doses. The results indicate that the absorption of orally administered inotodiol was moderate and variable. It might be attributed to the partial crystallization of inotodiol in the gastrointestinal fluid, resulting in a low oral bioavailability of inotodiol.

### 3.7. Anti-Sepsis Properties of Inotodiol

Inotodiol has previously been proven as a strong anti-allergic oxysterol due to the repression of mast cells [8]. Recently, strong inhibition of NF-kB signaling pathways in human dermal fibroblast cells by inotodiol was reported [5]. In this study, we evaluated the efficacy of inotodiol in preventing acute sepsis using different formulations. A strong corticosteroid, dexamethasone, was used as a positive control.

**Figure 7:**
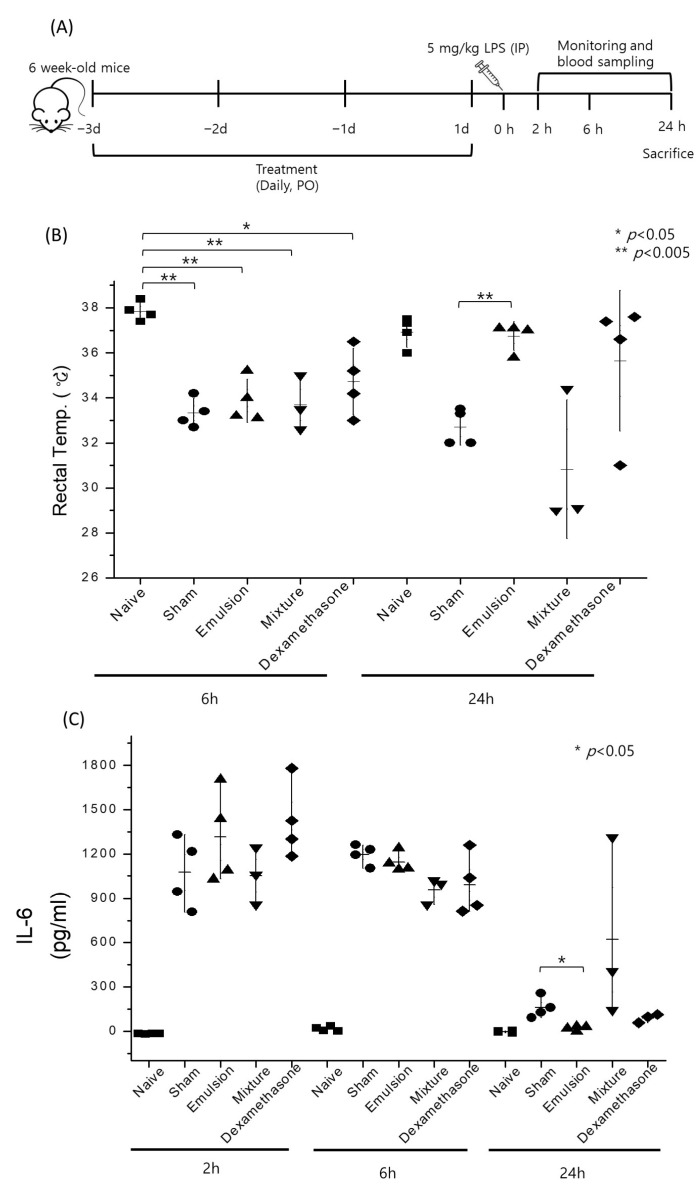
Evaluation of the anti-septic effects of inotodiol in different formulations. Schematic presentation of the acute sepsis model is shown (**A**). Rectal temperature (**B**) and IL-6 concentrations in serum (**C**) were measured to monitor overall symptoms and systemic inflammation levels.

After LPS injection, the rectal temperature of mice significantly decreased in all the sepsis groups at 6 h. However, it recovered in the inotodiol emulsion and dexamethasone groups at 24 h (Figure 7B). In the emulsion group, no diarrhea was observed, and the activities of mice were normal. Changes in the IL-6 level in serum were determined after the LPS injection. TNF-α and IL-1β levels in serum were measured at 24 h but were too low in every group to be compared (data not included). After LPS injection, IL-6 concentrations in serum greatly increased in all the groups at 2 h and 6 h. After 24 h, IL-6 levels decreased, but more significantly in the inotodiol emulsion group (*p* < 0.05) compared with the sham group. The dexamethasone group showed no statistical difference in IL-6 levels when compared with the sham group.

Activities of mice, rectal temperature, and diarrhea are used as indicators for overall symptoms of the acute sepsis model. TNF-α, IL-1β, and IL-6 are common proinflammatory cytokines that are monitored as sepsis biomarkers in mice. Especially, IL-6 is known as a biomarker of systemic inflammation. In this study, the inotodiol emulsion treatment successfully prevented the severe symptoms of acute sepsis, but the inotodiol mixture with Tween-80 did not. This result corresponds with the pharmacokinetics results of the new emulsion system of inotodiol. Intriguingly, the common inflammation biomarkers such as IL-6, TNF-α, and IL-1β were not strongly affected by the inotodiol treatment. As previously reported, inotodiol intensely inhibits the activation of mast cells, but not much information on other immune cells is known. To clarify the anti-septic effects of inotodiol, more studies on the molecular mechanism are essential. On the other hand, mast cells are deeply involved in the sepsis response of mice [45,46]. It is feasible that the fast recovery of the emulsion group was attributed to the repression of mast cells by inotodiol. In a further study, we will investigate the molecular mechanisms of the anti-septic effects of inotodiol.

## 4. Conclusions

This study has successfully developed a new method for high-purity inotodiol production from Chaga mushrooms. This method is more efficient than previous methods since the solvent system used for extraction and purification has been optimized. Moreover, the method can easily be scaled up to a larger scale, facilitating further research and development of inotodiol. In addition, the physicochemical properties of inotodiol were precisely elucidated and compared with cholesterol and lanosterol. Due to the difference in the C-22 hydroxyl group, inotodiol showed a distinct melting point and enthalpy, much higher than cholesterol and lanosterol. Therefore, the solubility of inotodiol in organic solvents was greatly limited, especially in *n*-hexane. DFT calculations further supported the experimental findings that the polarity of solvents significantly affected the solubility of inotodiol due to their strong effects on the hydroxyl groups at C-3 and C-22 of inotodiol. A microemulsion-based delivery system of inotodiol showed that the oral bioavailability (33–41%) greatly improved compared with the previous study (0.45%) [37]. In addition, the microemulsion system of inotodiol was more effective than the inotodiol mixture with Tween-80 in preventing acute sepsis. Intriguingly, the common inflammation biomarkers such as IL-6, TNF-α, and IL-1β were not strongly affected by the inotodiol treatment. The mechanisms of the protective effect of inotodiol against acute sepsis are not yet fully understood. Therefore, further studies on the molecular mechanisms of the anti-septic effects of inotodiol would shed more light on possible avenues for treating sepsis.

## Figures and Tables

**Table 2:** Pharmacokinetic parameters of inotodiol in mice plasma after oral administration and intraperitoneal administration.

Parameters	Units	Oral (8.0 mg/kg)	Oral (4.5 mg/kg)	Intraperitoneal (4.5 mg/kg)
k_a_	1/h	1.60 ± 1.29	0.77 ± 0.20	2.14 ± 1.32
t_1/2_k_a_	h	0.65 ± 0.34	0.95 ± 0.19	0.40 ± 0.14
T_max_	h	1.28 ± 0.48	1.44 ± 0.20	0.70 ± 0.17
C_max_	ng/mL	116.25 ± 43.54	88.05 ± 17.06	412.81 ± 155.12
AUC_0–24h_	ng·h/mL	489.45 ± 252.66	341.81 ± 86.77	827.20 ± 216.68
F	%	33.28	41.32	-

Note: k_a_, absorption rate constant; t_1/2_k_a_, absorption half-life; T_max_, time to reach C_max_; C_max_, maximum drug concentration in plasma; AUC, area under the plasma drug concentration curve from 0 to 24 h; F, oral bioavailability.

## Data Availability

Data are contained within the article and Supplementary Materials.

## Supplementary Materials

The following supporting information can be downloaded at: https://www.mdpi.com/article/10.3390/antiox12020447/s1, Supplementary Data S1: Table S1: The mobile phase gradients and HPLC-ELSD instrument conditions. Supplementary Data S2: Table S2: The mobile phase gradients and HPLC-MS/MS instrument conditions. Table S3: The positive mode and MS/MS conditions. Supplementary Data S3: Figure S1: The solubility of inotodiol in ethanol, methanol, acetonitrile, acetone, and *n*-hexane at different temperatures. Supplementary Data S4: Figure S2: The separation of inotodiol using methanol and acetonitrile as a mobile phase. Supplementary Data S5: Figure S3: The proposed fragmentation pathway of inotodiol in the positive ion mode. Supplementary Data S6: Inotodiol NMR data. Supplementary Data S7: Table S4: Bond lengths in the optimized geometry of inotodiol, cholesterol, and lanosterol in different solvents. Figure S3: Selected bond lengths (Å) of optimized geometries of cholesterol in ethanol (polar protic solvent), acetone (polar aprotic solvent), and *n*-hexane (nonpolar solvent) compared to in the gas phase. Figure S4: Selected bond lengths (Å) of optimized geometries of lanosterol in ethanol (polar protic solvent), acetone (polar aprotic solvent), and *n*-hexane (nonpolar solvent) compared to in the gas phase. Figure S5: Selected bond lengths (Å) of optimized geometries of inotodiol in ethanol (polar protic solvent), acetone (polar aprotic solvent), and *n*-hexane (nonpolar solvent) compared to in the gas phase. Figure S6: HPLC chromatogram of Chaga extract and inotodiol after purification.
