# Supplementary material for: Isolation, Physicochemical Characterization, and Biological Properties of Inotodiol, the Potent Pharmaceutical Oxysterol from Chaga Mushroom
Source: Antioxidants (Basel). 2023 Feb 10;12(2):447. doi: 10.3390/antiox12020447 (PMC9952744; doi:10.3390/antiox12020447)
Supplement: Supplementary file 1 [file antioxidants-12-00447-s001.zip › antioxidants-2188723-supplementary.pdf]

Supplementary Data S1

Table S1. The mobile phase gradients and HPLC-ELSD instrument conditions

The mobile phase gradients			HPLC-ELSD conditions				
Time (min)	A (%) DW	B (%) Methanol	Injection volume (μL)	Flow rate (mL/min)	Gas flow L/min	Drift-tube temperature (°C)	Column temperature (°C)
0	20	80	10	1	3	90	50
5.0	5	95					
10.0	5	95					
10.1	0	100					
20.0	0	100					
20.1	20	80					
25	20	80					

Supplementary Data S2

Table S2. The mobile phase gradients and HPLC-MS/MS instrument conditions

The mobile phase gradients			HPLC-MS/MS conditions			
Time (min)	A (%) DW	B (%) Methanol	Injection volume (μL)	Flow rate (mL/min)	Column temperature (°C)	Mode
0	25	75	2	0.2	40	Positive
2	25	75				
6	10	90				
8	3	97				
16	0	100				
17	25	75				
20	25	75				

Table S3. The positive mode and MS/MS conditions

Positive mode conditions						
Dry gas temperature (°C)	Dry gas flow rate (L/min)	Nebulizer pressure (psi)	Sheath gas temperature (°C)	Sheath gas flow rate (L/min)	Capillary voltage (V)	Nozzle voltage (V)
270	10	40	300	11	3500	500
MS/MS transition conditions						
Analysis	Q1 (m/z)	Q3 (m/z)	Dwell	Fragment Voltage (V)	Collision energy (V)	Cell Accelerate (V)
Inotodiol	425.4	247.1	150	135	10	7
Inotodiol	425.4	229.1	150	135	18	7

Supplementary Data S3

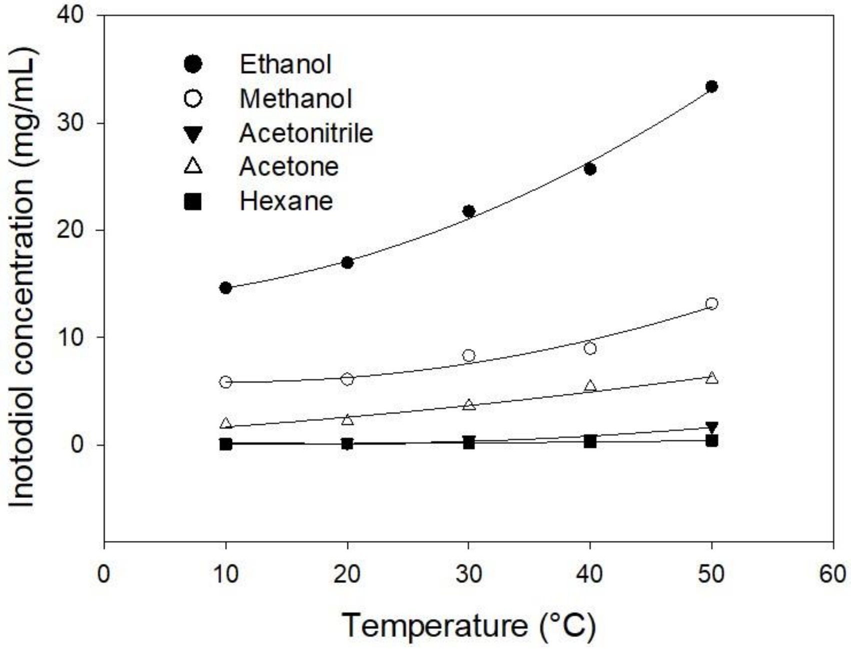

Figure S1. The solubility of inotodiol in ethanol, methanol, acetonitrile, acetone, and n-hexane at different temperatures.

Supplementary Data S4

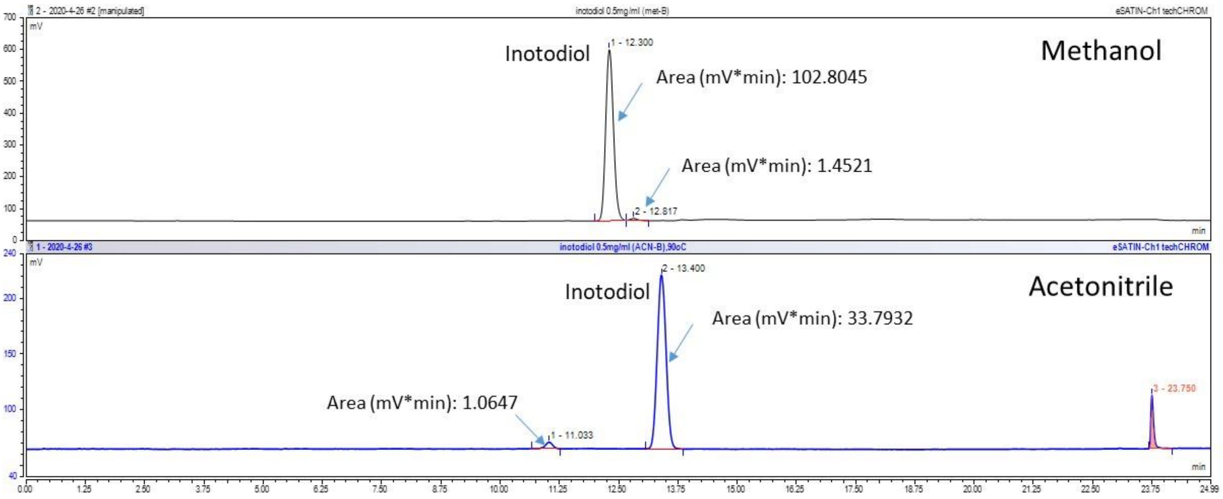

Figure S2. The separation of inotodiol using methanol and acetonitrile as a mobile phase.

Supplementary Data S5

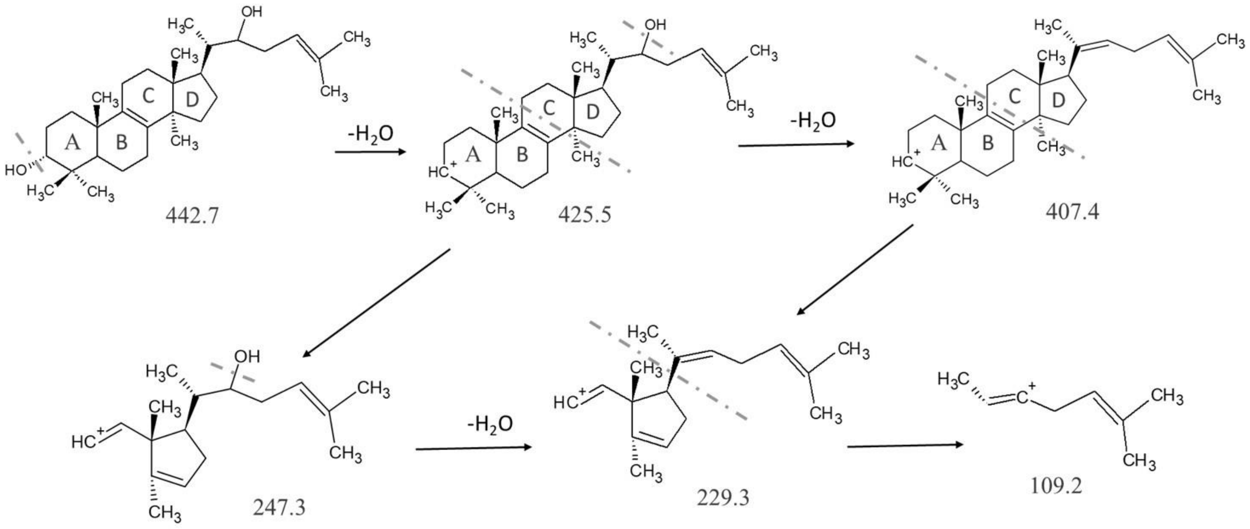

Figure S3. The proposed fragmentation pathway of inotodiol in the positive ion mode.

Supplementary Data S6

Inotodiol NMR data:

^{1}H NMR (600 MHz, $CDCl_3$): **δ** 5.10 (1H, t, J =7.2 Hz, 24-H), 3.59 (1H, m, J =4.035 Hz, 22-H), 3.15 (1H, dd, J =11.64 Hz, 3-H), 0.64 (3H, s, 18-Me), 0.73 (3H, s, 29-Me), 0.79 (3H, s, 30-Me), 0.86 (3H, d, J =6.58 Hz, 21-Me), 0.91 (3H, d, J =10.53 Hz, 28-Me), 0.97 (3H, dd, J =12.82 Hz 19-Me), 1.57 (3H, s, 26-Me), 1.66 (3H, s, 27-Me), 1.96 (1H, m, 20-H), 1.46 (1H, m, 17-H), 1.17 (1H, m, 5-H).

^{13}C NMR (150 MHz, $CDCl_3$): **δ** 135.16 (C-25), 134.58 (C-9), 134.20 (C-8), 121.25 (C-24), 78.98 (C-3), 73.38 (C-22), 50.39 (C-5), 49.40 (C-14), 47.26 (C-17), 44.86 (C-13), 41.69 (C-20), 38.89 (C-4), 37.03 (C-10), 35.58 (C-1), 30.96 (C-15, C-16), 29.12 (C-7), 27.96 (C-29), 27.84 (C-2), 27.24 (C-23), 26.51 (C-12), 25.97 (C-26), 24.29 (C-30), 21.00 (C-11), 19.13 (C-6), 18.24 (C-19), 17.97 (C-27), 15.68 (C-18), 15.40 (C-28), 12.60 (C-21).

Supplementary Data S7

Table S4. Bond lengths in the optimized geometry of inotodiol, cholesterol, and lanosterol in different solvents.

Bonds lengths (Å)								
	**Inotodiol**				**Cholesterol**		**Lanosterol**	
	C3-O31	O31-H32	C22-O33	O33-H34	C3-O31	O31-H32	C3-O31	O31-H32
Gas	1.419	0.957	1.424	0.958	1.417	0.958	1.419	0.957
Acetonitrile	1.423	0.96	1.428	0.96	1.421	0.96	1.423	0.96
Acetone	1.423	0.96	1.427	0.961	1.421	0.961	1.423	0.96
Methanol	1.428	0.96	1.432	0.961	1.426	0.961	1.428	0.96
Dichloromethane	1.423	0.96	1.427	0.961	1.421	0.961	1.423	0.96
Ethanol	1.427	0.96	1.43	0.961	1.424	0.961	1.427	0.96
n-Hexane	1.421	0.958	1.425	0.959	1.419	0.959	1.421	0.958
**Elongation (Å)**								
	**Inotodiol**				**Cholesterol**		**Lanosterol**	
	C3-O31	O31-H32	C22-O33	O33-H34	C3-O31	O31-H32	C3-O31	O31-H32
Acetonitrile	0.004	0.003	0.004	0.002	0.004	0.002	0.004	0.003
Acetone	0.004	0.003	0.003	0.003	0.004	0.003	0.004	0.003
Methanol	0.009	0.003	0.008	0.003	0.009	0.003	0.009	0.003
Dichloromethane	0.004	0.003	0.003	0.003	0.004	0.003	0.004	0.003
Ethanol	0.008	0.003	0.006	0.003	0.007	0.003	0.008	0.003
n-Hexane	0.002	0.001	0.001	0.001	0.002	0.001	0.002	0.001

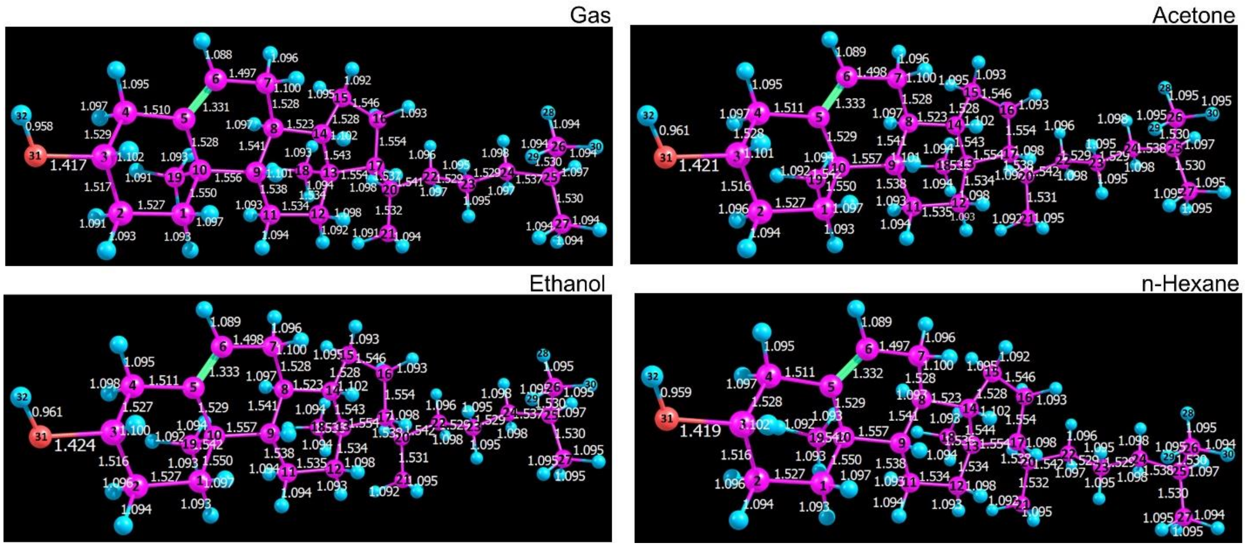

Figure S3. Selected bond lengths (Å) of optimized geometries of cholesterol in ethanol (polar protic solvent), acetone (polar aprotic solvent), and *n*-hexane (nonpolar solvent) compared to in the gas phase. Carbon, oxygen, and hydrogen are given in pink, red, and blue balls.

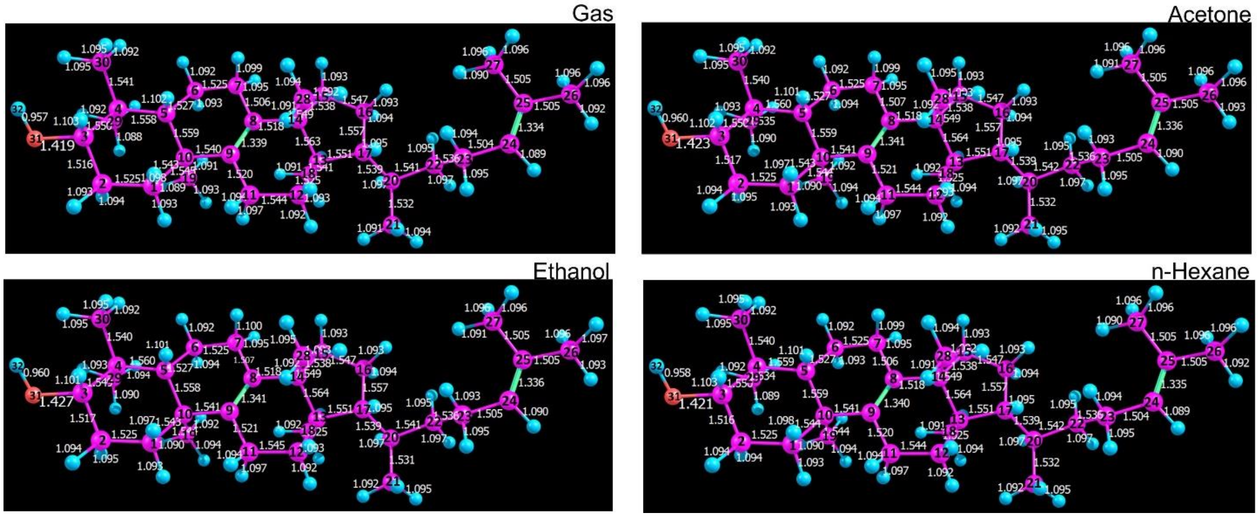

Figure S4. Selected bond lengths (Å) of optimized geometries of lanosterol in ethanol (polar protic solvent), acetone (polar aprotic solvent), and *n*-hexane (nonpolar solvent) compared to in the gas phase. Carbon, oxygen, and hydrogen are given in pink, red, and blue balls.

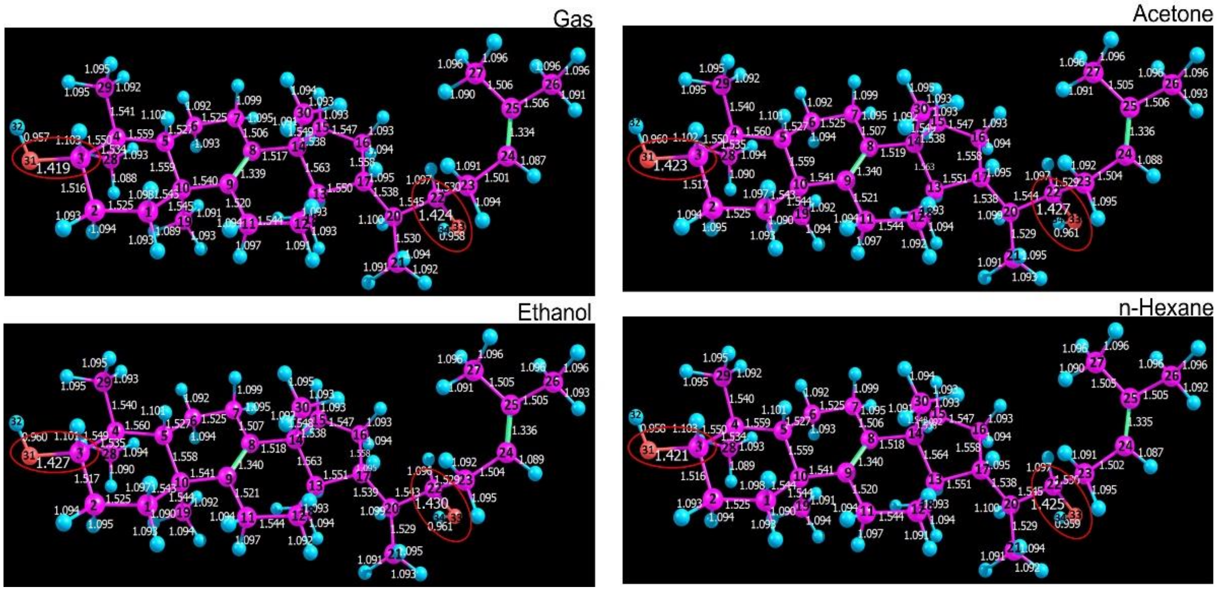

Figure S5. Selected bond lengths (Å) of optimized geometries of lanosterol in ethanol (polar protic solvent), acetone (polar aprotic solvent), and *n*-hexane (nonpolar solvent) compared to in the gas phase. Carbon, oxygen, and hydrogen are given in pink, red, and blue balls.

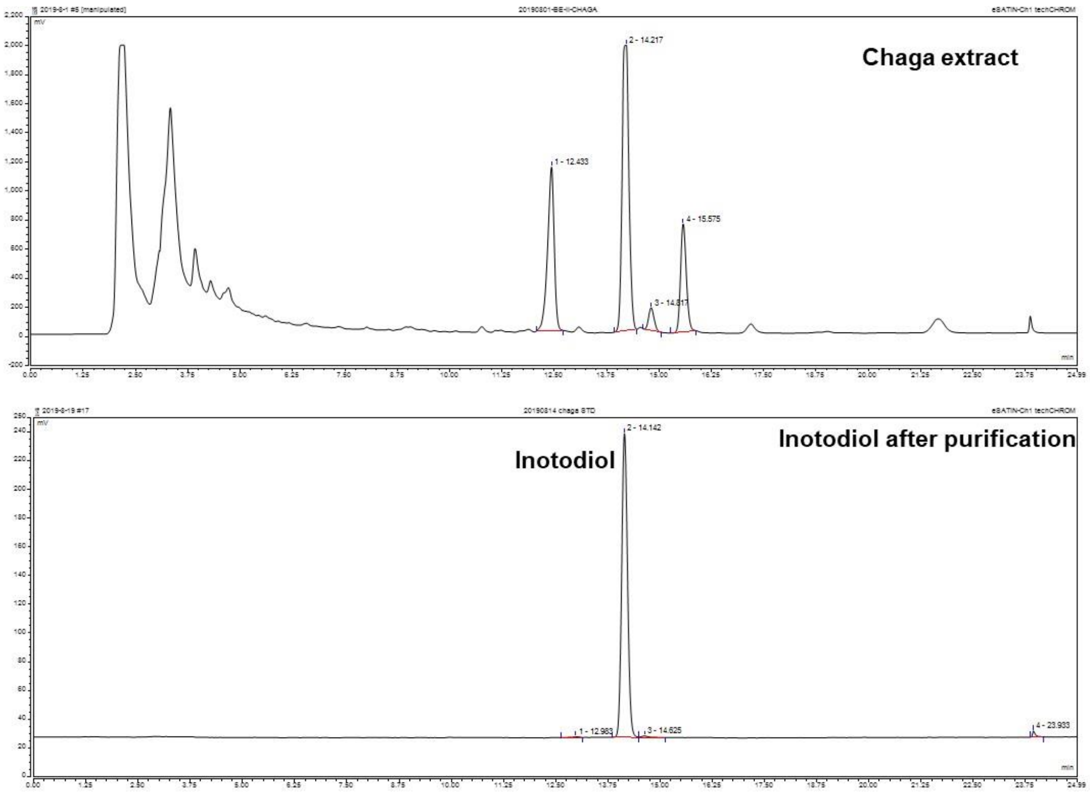

Figure S6. HPLC chromatogram of Chaga extract and inotodiol after purification.
